# Iron(II) Spin Crossover Complexes with 4,4′-Dipyridylethyne—Crystal Structures and Spin Crossover with Hysteresis

**DOI:** 10.3390/molecules25030581

**Published:** 2020-01-29

**Authors:** Katja Dankhoff, Charles Lochenie, Birgit Weber

**Affiliations:** Department of Chemistry, Inorganic Chemistry IV, University of Bayreuth, Universitätsstr. 30, 95447 Bayreuth, Germany; katja.dankhoff@uni-bayreuth.de (K.D.); locheniec@gmail.com (C.L.)

**Keywords:** spin crossover, iron, Schiff base ligand, hysteresis, cooperative interactions

## Abstract

Three new iron(II) 1D coordination polymers with cooperative spin crossover behavior showing thermal hysteresis loops were synthesized using N_2_O_2_ Schiff base-like equatorial ligands and 4,4′-dipyridylethyne as a bridging, rigid axial linker. One of those iron(II) 1D coordination polymers showed a 73 K wide hysteresis below room temperature, which, upon solvent loss, decreased to a still remarkable 30 K wide hysteresis. Single crystal X-ray structures of two iron(II) coordination polymers and *T*-dependent powder XRD patterns are discussed to obtain insight into the structure property relationship of those materials.

## 1. Introduction

Iron(II) spin crossover (SCO) complexes belong to an interesting class of materials which can be switched between the paramagnetic high spin state (HS, *S* = 2), and the diamagnetic low spin state (LS, *S* = 0) by external stimuli such as temperature, pressure, or light irradiation [1,2,3,4,5,6,7,8,9,10,11,12,13,14,15,16,17,18,19,20,21]. This transition causes changes in the optical, vibrational, magnetic, and structural properties of the complexes and can therefore be monitored by many different techniques, such as magnetic susceptibility measurements, Mössbauer spectrometry, or single crystal or powder X-ray diffraction at different temperatures [22,23]. Due to the versatile possibilities to trigger and follow the spin state changes, SCO active compounds have a broad field of potential applications. As they often show a significant change of color upon spin transition (ST), use as temperature or pressure sensors with an easy optical readout is possible [20,24,25,26]. Other discussed applications for SCO complexes are data storage, displays [27,28,29], or more recently as molecular actuators [30]. From the many possible types of SCO (abrupt, gradual, stepwise, incomplete) SCO complexes with a thermal hysteresis around room temperature are most suitable for this [31,32,33,34,35,36,37,38]. Consequently, the molecular and environmental requirements for the observation of spin crossover with hysteresis are still being investigated very intensely. By now, there are some well-established models to explain the interplay of changes in the molecular structure, crystal packing parameters, and the observation of thermal hysteresis loops [18]. Nevertheless, there is still a lot to be learned for the different substance families to further improve this understanding. The detailed understanding of the interplay of packing parameters and observed thermal hysteresis loop is very important for potential applications, especially if the complexes are to be incorporated into composite materials, for examples as nanoparticles [39,40,41,42,43,44,45], or if additional properties are to be incorporated without losing the cooperative spin crossover properties [46,47,48,49,50].

An additional aspect to be considered is that the SCO is highly dependent on the solvent included in the crystal packing and the choice of the solvent plays a very important role for synthesizing complexes with SCO behavior. Solvent molecules included in the crystal packing can influence the packing as many solvents can form hydrogen bonds, which have been shown to enhance cooperativity in the SCO behavior of the complex [34]. The loss of solvent upon drying can change the SCO behavior significantly, thus if very rigid ligands are used, a reversible binding of solvent can be used for solvent sensing [25,51,52].

Recently, our group has reported a large thermal hysteresis for an iron(II) complex with Schiff base-like equatorial ligands and *N*-(pyrid-4-yl)isonicotinamide as an axial linker, where the amount and type of incorporated solvent was highly important for the width of the hysteresis [53]. Other 1D coordination polymers with rigid linkers, such as 4,4′-bipyridine, have been shown to present hysteretic SCO behavior [54,55,56], whereas more flexible linkers often resulted in a loss of the cooperative effects and steps in the transition curve [57,58,59,60]. Here we present three new 1D chain coordination polymers with 4,4′-dipyridylethyne (bpey) as a rigid, axial ligand with varying contents of the solvent methanol, that display a different hysteretic SCO behavior depending on the substituents on the equatorial linker and the included amount of solvent molecules. Hoffmann clathrate complexes with bpey as a ligand have been reported that also show different magnetic behaviors depending on the amount of solvent. An interesting aspect of the bpey ligand is its rather rigid nature, which, in concert with its thread-like appearance, may lead to the formation of larger voids needed for solvent inclusion. 

## 2. Results

### 2.1. Synthesis

**Scheme 1:**
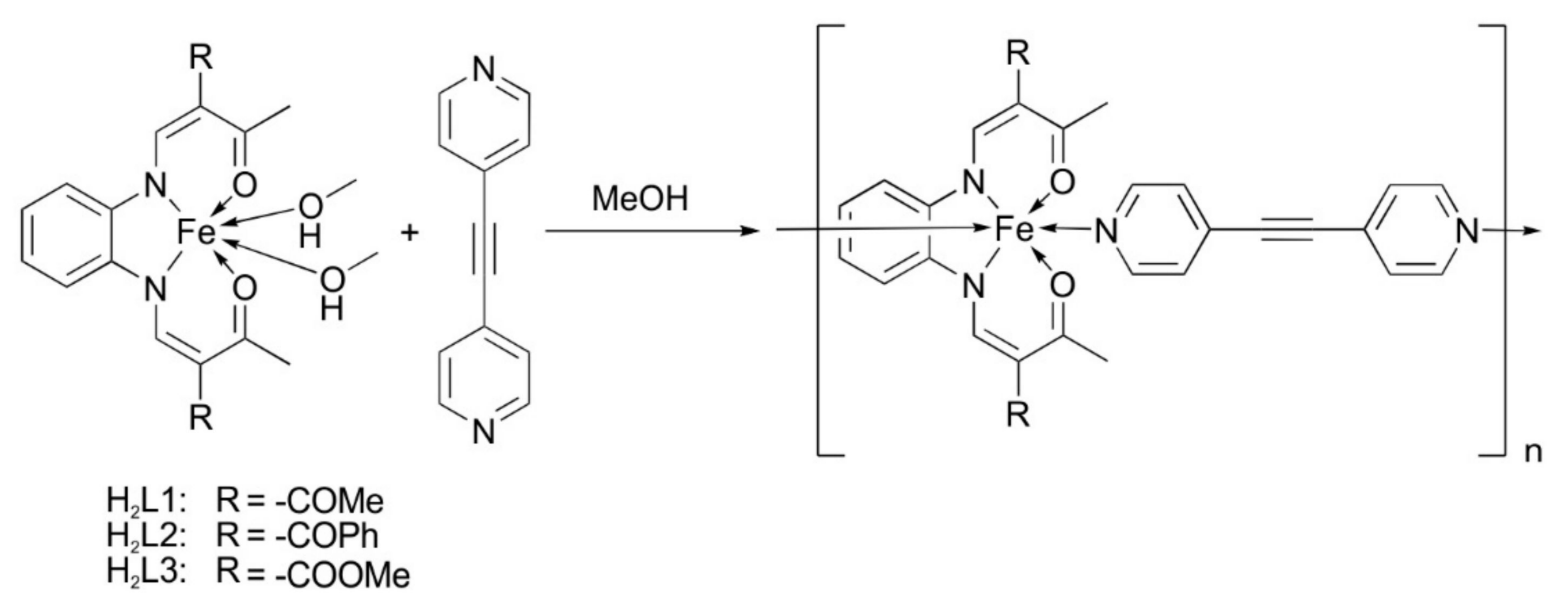
General synthesis procedure for the presented iron(II) coordination polymers with 4,4′-dipyridylethyne (bpey).

The general synthesis for the iron(II) complexes is based on a ligand exchange reaction between the axial methanol ligands of the already reported iron(II) precursor complex [57,61,62,63] and the axial, bidentate ligand bpey [64,65]. Therefore the precursor complex and bpey were dissolved in MeOH and heated to reflux for 1 h. Slow diffusion crystallization setups at room temperature were made to obtain crystals suitable for X-ray structure analysis. Scheme 1 displays the general synthesis pathway for the iron(II) complexes and Table 1 gives an overview of all synthesized iron(II) complexes and the included solvent content. The prepared iron(II) complexes all crystallized as very dark, fine-crystalline material and were analytically pure, however they contained varying solvent contents according to elemental analysis and mass spectrometry in all cases. Coordination polymers were obtained in all cases as usually observed for this type of compounds [66,67,68,69]. The solvent contents can be controlled for **1** and **3** by the reaction temperature, where higher reaction temperatures (precipitation while refluxing) usually lead to a lower amount of solvent molecules included in the crystal packing.

### 2.2. Crystal Structure Analysis

Single crystals of suitable quality for X-ray structure analysis were obtained for **1b** and **3b** by using slow diffusion techniques at room temperature between solutions of the respective iron(II) precursor and bpey in methanol at room temperature.

**Figure 1:**
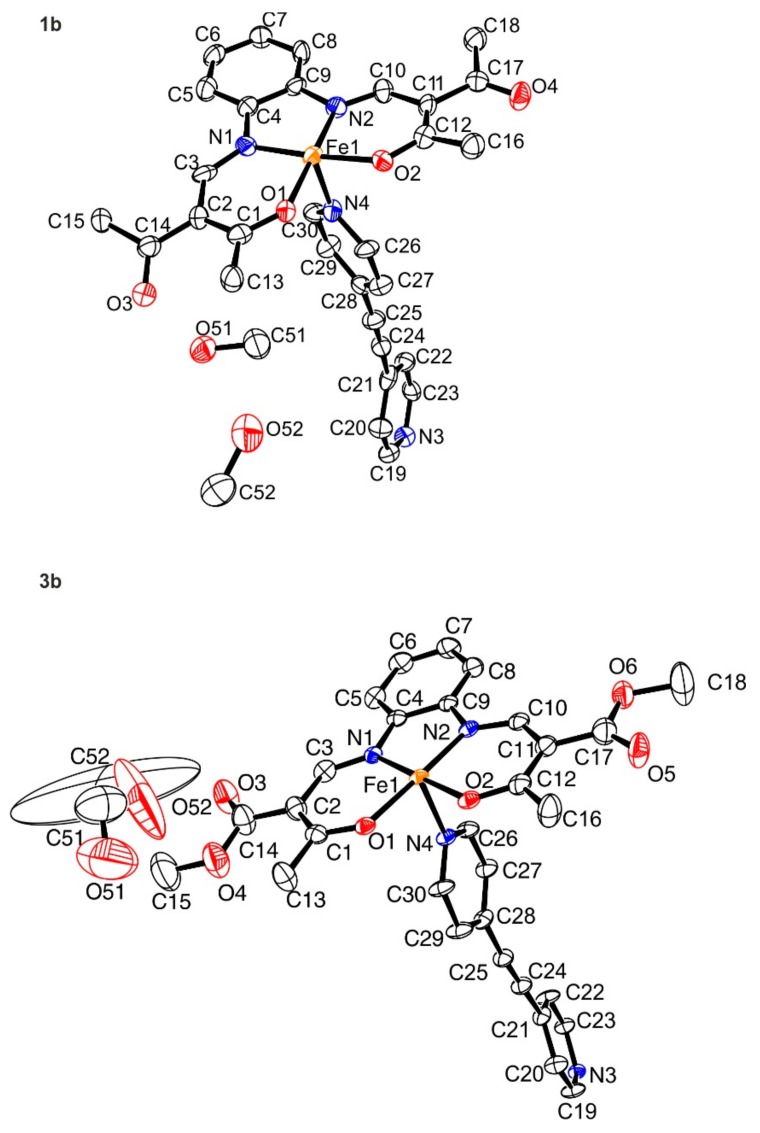
Structure of the asymmetric unit of **1b** (**top**) and **3b** (**bottom**). Please note that for **1b** only a structural motive was obtained. For **3b**, ellipsoids are drawn at a 50% probability level. Hydrogen atoms and disordered atoms were omitted for clarity.

The coordination polymers **1b** and **3b** crystallize in the triclinic space group *P*−1 and the data for the crystal structure determination were collected at 140 K (**1b**) and 133 K (**3b**). The crystallographic data are given in Table S1 and the asymmetric units are depicted in Figure 1. Each asymmetric unit contains the monomeric unit of the respective coordination polymer and two non-coordinating methanol molecules. Due to the low quality of the single crystals of **1b** the obtained data do not allow a full structure refinement and consequently only the general structural motif will be discussed. No conclusions regarding bond lengths, angles, and/or intermolecular interactions will be drawn. The iron(II) centers of both compounds are in an octahedral coordination environment, and the bidentate ligand bpey links the metals to form infinite one-dimensional chains. In the case of compound **3b** a disorder of the substituents of the chelate cycle (O5, O6, C18) into two positions can be observed (Figure S1).

**Figure 2:**
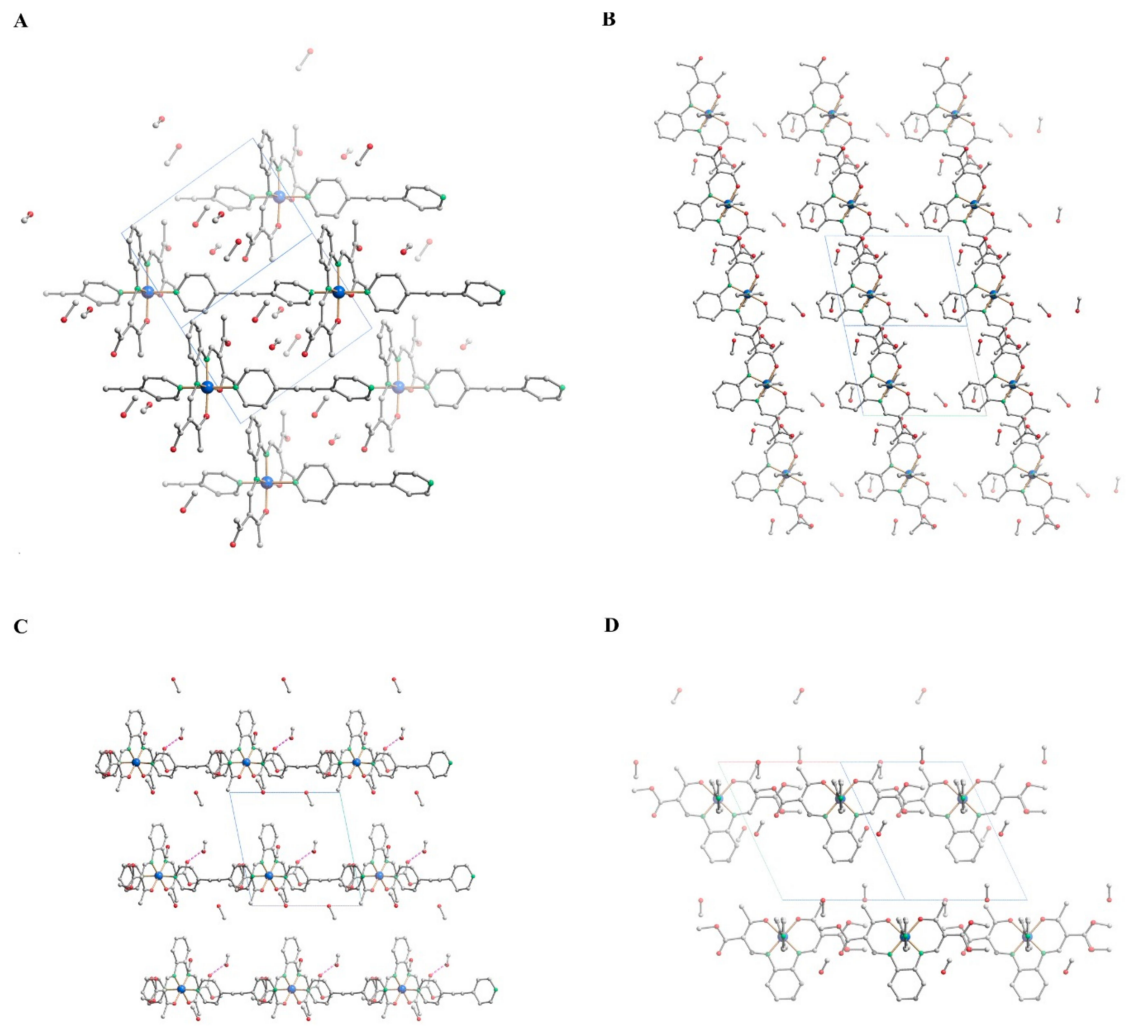
Molecular packing of 1b (A, along [011] and B, along [−1−10]), and 3b (C, along [010] and D, along [−110]) in the crystal. Hydrogen atoms not involved in hydrogen bonds were omitted for clarity. Hydrogen bonds are drawn as pink, dashed lines.

Selected bond lengths and angles for **3b** are given in Table 2. The average bond lengths are with Fe-N_eq_ 1.895 Å, Fe-N_ax_ 1.991 Å, and Fe-O 1.931 Å in the typical region for iron(II) LS complexes of this ligand type [70,71]. The O_eq_–Fe–O_eq_ angle of 87.08(11) also indicates that the metal center is in the LS state. The >C-C≡C angle of 175.6° (**3b**) indicates a slight bending of the ligand bpey. Analysis of the polymeric structures (Figure 2) reveals an infinite one-dimensional chain along [−1−10] (**1b**) and [−110] (**3b**). The torsion angle between the pyridyl rings of **3b** is relatively small with an average value of 17.8°. One of the two included methanol molecules of the structure is involved in a hydrogen bond to a carbonyl atom of the complex (O52–H52⋯O3). A second observed hydrogen bond explains the bending of the ligand bpey. C26–H26 is a donor for this hydrogen bond to the carbonyl atom O3 of a neighboring equatorial ligand. This leads to the formation of pairs of two chains of the coordination polymer, leading to a ladder-like arrangement in the crystal packing. Details of the hydrogen bonds are given in Table 3. The molecular packing with its hydrogen bond pattern is given in Figure 2. 

### 2.3. Magnetism

**Figure 3:**
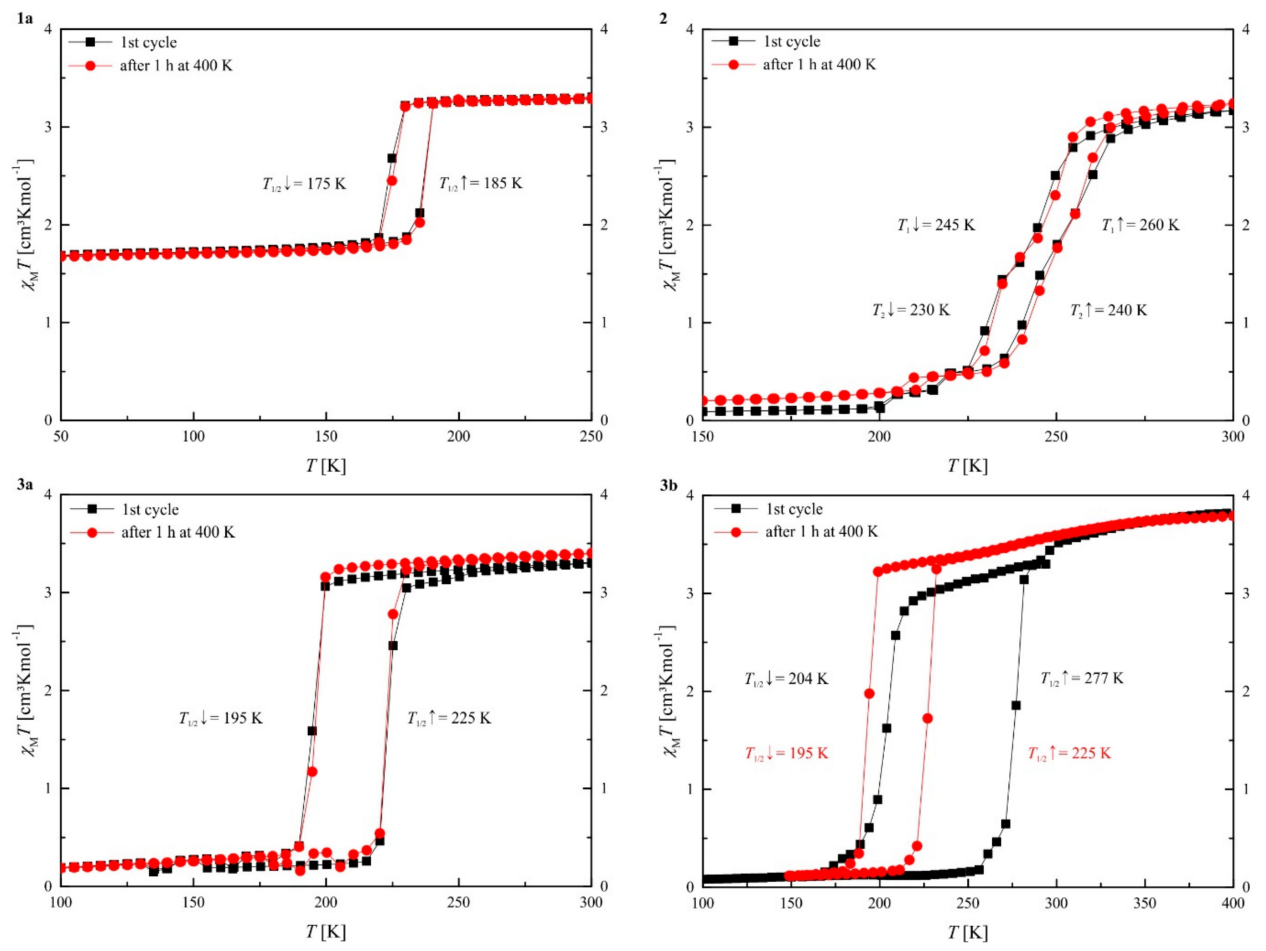
Plots of the *χ*_M_*T* product vs. *T* for compounds **1a**, **2**, **3a**, and **3b**. Black squares represent the first cooling and heating cycle and red circles correspond to the cooling and heating cycle after annealing.

Magnetic susceptibility measurements were performed with an applied field of 5000 G on a SQUID magnetometer in varying temperature ranges to follow the ST. After initial measurements in the sweep mode (Figure S2) with a sweep rate of 5 Kmin^−1^, the plots depicted here were obtained in the settle mode with an approximate average sweep rate of 0.5 Kmin^−1^. No significant differences between the two measurement velocities were observed, indicating the absence of kinetic trapping effects [37,60,72,73]. The plots of the *χ*_M_*T* product versus *T* for the complexes are displayed in Figure 3, the values are summarized in Table 4. As both elemental analysis and the results from single crystal X-ray structure analysis indicated the presence of methanol molecules in the crystal packing for **2**, **3a** and **3b**, after the initial measurements between room temperature and 50 K, the samples were heated to 400 K in the cavity of the SQUID magnetometer in order to remove the solvent and a second measurement cycle between 400 K and 50 K was made.

Complex **1a** shows a half-complete ST with a 10 K wide hysteresis with *T*_1/2_↓ = 175 K and *T*_1/2_↑ = 185 K. The value of the *χ*_M_*T* product is 3.29 cm^3^Kmol^−1^ at 250 K, which is in the typical region for iron(II) HS. After the ST the value of the *χ*_M_*T* product is 1.69 cm^3^Kmol^−1^ at 50 K, which is almost exactly half of the value before the ST. This indicates that the compound has two different iron(II) sites, where one of them is SCO active and the other one is not. Room temperature Mössbauer spectroscopy was used to investigate if two inequivalent iron sites are present in the crystal packing. As shown in Figure S3, only one quadrupole split doublet is observed that is very symmetric and has a narrow line width. Thus it appears that the inequivalent nature of the iron centers is generated during the spin transition, a phenomenon already observed for similar iron(II) coordination polymers [58,74]. After heating to 400 K for one hour the ST has not changed, in line with the absence of solvent molecules in the crystal packing. It still shows the same hysteresis, and no changes in the values of the *χ*_M_*T* product are observed. Magnetic measurement on the single crystals **1b** showed the same magnetic behavior as for the powder sample. This indicates that the crystals lose their solvent already during sample preparation for the SQUID measurements. A further indication of this is that only a single sort of iron centers is observed in the motif obtained from X-ray structure analysis at 140 K. According to the magnetic measurements, at this temperature two different iron(II) sites, one HS and one LS, should be present in the asymmetric unit of the crystal structure. Please note that it cannot be ruled out that **1a** initially has the same solvent contents/structure as **1b** but quickly loses the solvent upon sample preparation due to the smaller crystallite size, thus appearing solvent-free. The plot of the *χ*_M_*T* product versus *T* is given in the Supplementary Materials, Figure S2.

Magnetic measurements of complex **2** reveal a stepwise ST with hysteresis below room temperature. For the first step a 15 K wide hysteresis is observed with *T*_1_↓ = 245 K and *T*_1_↑ = 260 K. The second step takes place right after the first one and has a 10 K wide hysteresis with *T*_2_↓ = 230 K and *T*_2_↑ = 240 K. The value of the *χ*_M_*T* product is with 3.17 cm^3^Kmol^−1^ at 300 K in the region for iron(II) HS. After the ST the value of the *χ*_M_*T* product has reached 0.09 cm^3^Kmol^−1^ at 150 K, a typical value for iron(II) LS. As the two steps are very close together it is difficult to determine a *χ*_M_*T* value for the plateau region. It appears to be in a region where about 50% of the molecules did switch the spin state. However, as for compound **1a**, the room temperature Mössbauer spectrum shows only one quadrupole split doublet that is very symmetric and characteristic for iron(II) complexes of this ligand type in the HS state. After heating to 400 K for one hour the ST did not change significantly; only minor changes in the values of the *χ*_M_*T* product can be found. Thus, the loss of solvent molecules in the SQUID cavity appears to have no impact on the SCO properties or the methanol molecules were lost during sample preparation as in the case of **1**.

The complex **3a** shows a 30 K wide hysteresis with *T*_1/2_↓ = 195 K and *T*_1/2_↑ = 225 K. The values of the *χ*_M_*T* products are with 3.31 cm^3^Kmol^−1^ at 300 K are typical for iron(II) HS and with 0.19 cm^3^Kmol^−1^ at 100 K are typical for iron(II) LS. After heating the sample to 400 K for one hour the ST characteristics do not change; only the value of the *χ*_M_*T* product at 300 K increases to 3.40 cm^3^Kmol^−1^, while the value of the *χ*_M_*T* product in the LS state remains the same. Single crystals of **3b** were collected from the mother liquor and directly measured. In the first cooling and heating cycle the crystals show a 73 K wide hysteresis with *T*_1/2_↓ = 204 K and *T*_1/2_↑ = 277 K. The value of the *χ*_M_*T* product at 400 K is with 3.79 cm^3^Kmol^−1^, slightly too high for pure iron(II) HS; this can be explained by measuring the “wet” crystals, which makes it difficult to define the exact mass of the sample measured. Partial oxidation of the sample can be ruled out as the value for the *χ*_M_*T* product at 100 K is 0.08 cm^3^Kmol^−1^ in the typical region for iron(II) LS. After heating to 400 K for one hour the sample loses this wide hysteresis and shows the same magnetic behavior as complex **3a**, with only minor differences in the values of the *χ*_M_*T* product. 

### 2.4. Powder X-Ray Diffraction Analysis

**Figure 4:**
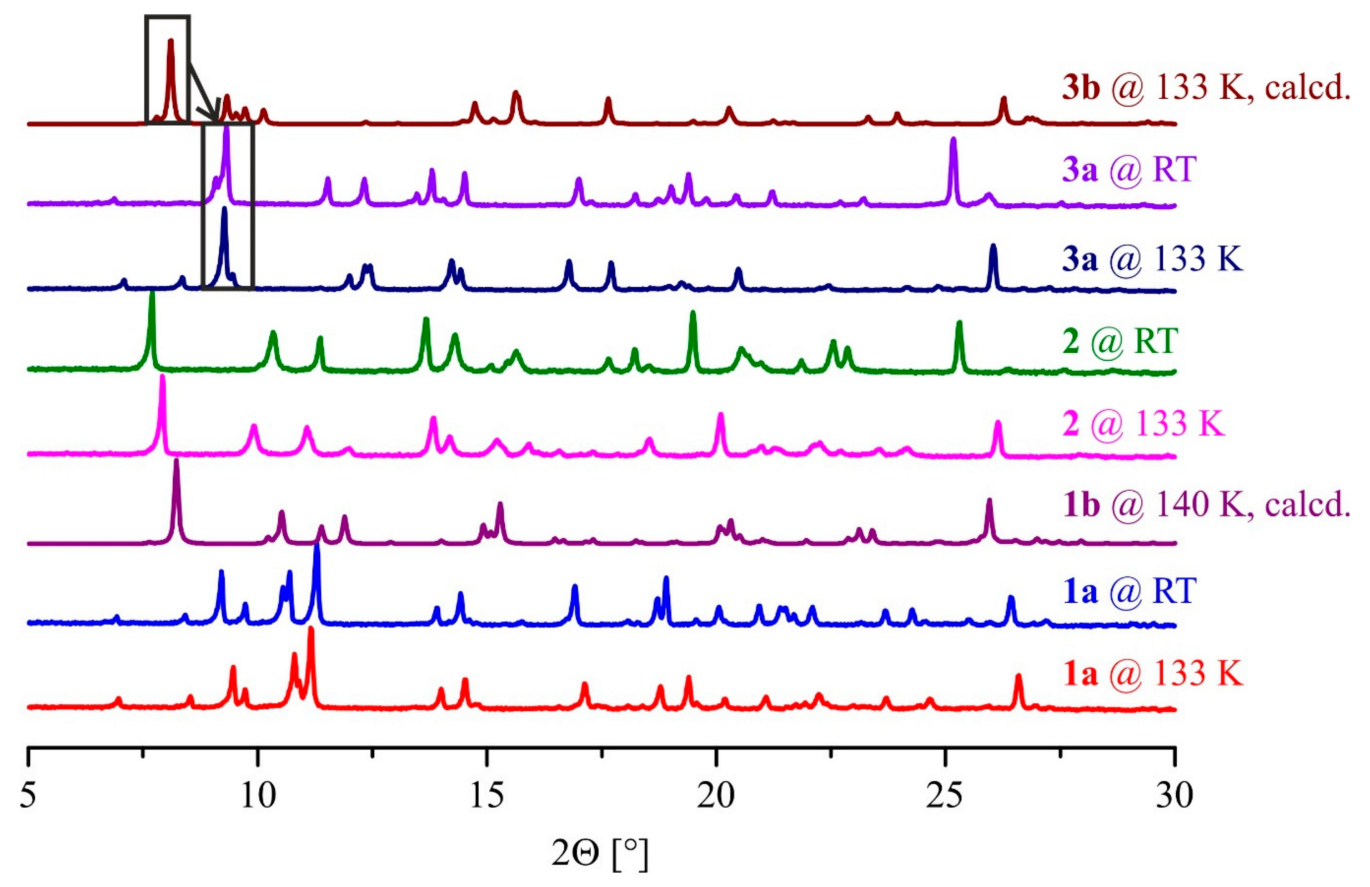
Powder X-ray diffraction patterns of **1a**, **1b**, **2**, **3a**, and **3b** at the indicated temperatures.

Powder X-ray diffraction on all samples was performed at various temperatures that were found diagnostic for the magnetic properties of the complexes. The measured and calculated patterns of the complexes **1**–**3** are shown in Figure 4. The patterns of **1a** at 133 K and RT are very similar showing only small shifts of the reflections. According to Bragg’s law (nλ= 2d sinΘ; n = 1, λ = 1.541 Å, d = interplanar distance, Θ = scattering angle), a shift of the reflections to higher 2Θ values indicates a shortening of distances which would be in line with the change from HS to LS iron(II). The powder pattern calculated from the single crystal data of **1b**, on the other hand, is very different from the measured powder patterns of **1a**. The crystal of the coordination polymer (**1b**) includes two molecules of methanol per iron(II) center that are not present in the isolated powder **1a** according to elemental analysis. The data indicate that there are some pronounced differences in the crystal packing of **1a** and **1b**. The powder patterns at the different temperatures (133 K and RT) of **2** are very similar with only small changes that can, once again, be explained by small changes of the bond lengths from LS to HS iron(II), leading to differences in the crystal packing at the different temperatures. The powder diffraction patterns of **3a** measured at different temperatures (133 K and RT) are quite different, which indicates a phase transition during the SCO. The calculated powder diffraction pattern of **3b** is completely different compared to the measured one of **3a**. As for **1a**/**1b**, the amount of solvent included in the single crystals (two molecules of methanol per iron(II) center) and the fine-crystalline powder (no solvent included) differs significantly. This could cause pronounced structural changes and the observed differences in the diffraction patterns. Again, those changes are in good agreement with the different behavior observed in the magnetic measurements.

### 2.5. Thermal Analysis

**Figure 5:**
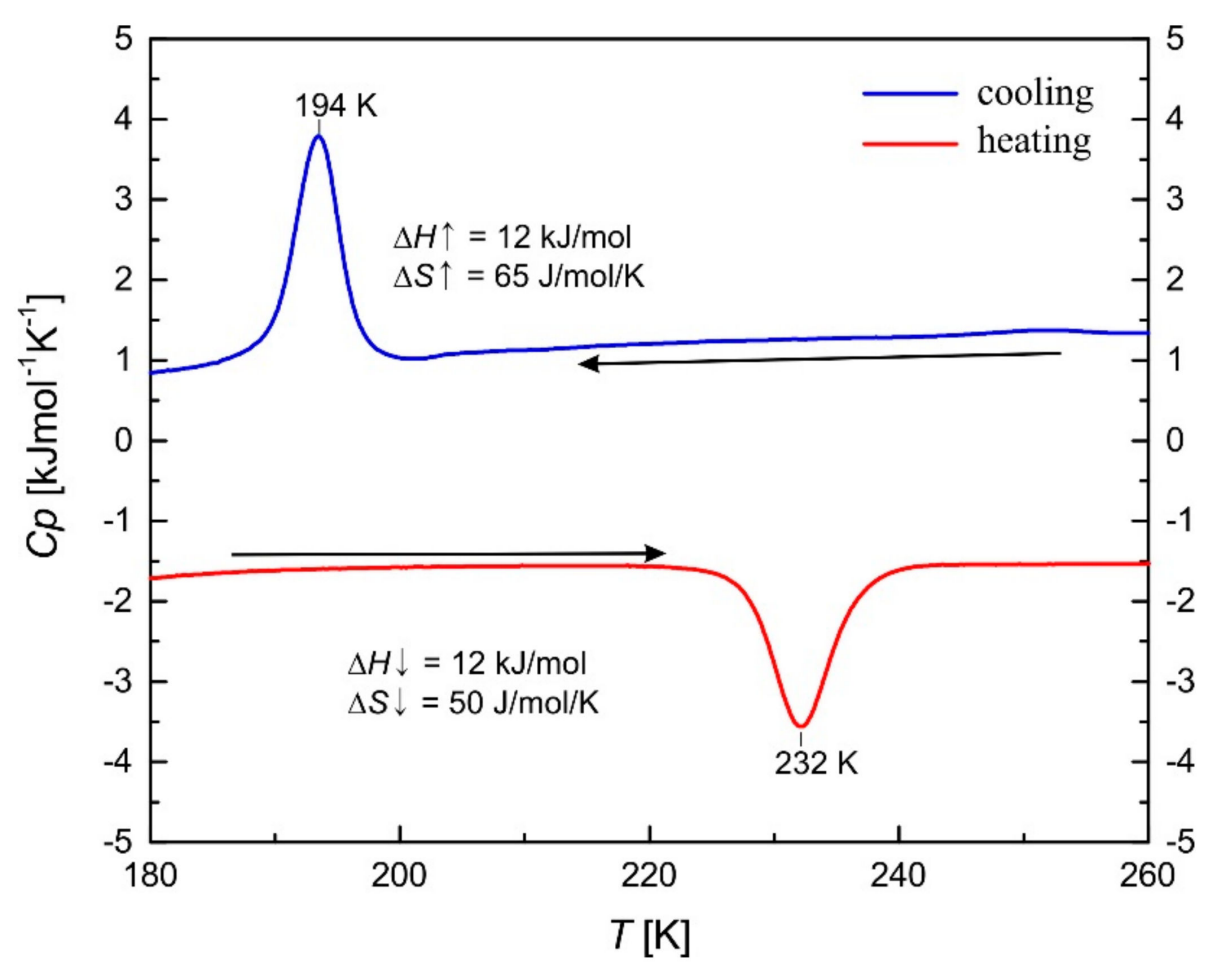
Differential scanning calorimetry (DSC) measurement of **3a**. Measured enthalpy and entropy values are given in the cooling (blue line) and heating (red line) modes (5 K/min).

Differential scanning calorimetry (DSC) was performed to further characterize the ST of **3a** and to track possible phase transitions that can occur during SCO (Figure 5). Upon cooling a sharp peak for the exothermic transition from the HS to the LS state is observed at 194 K with Δ*H* = 12 kJ/mol and Δ*S* = 65 J/molK. Upon warming a sharp peak for the endothermic transition from the LS to the HS state is observed at 232 K with Δ*H* = 12 kJ/mol and Δ*S* = 50 J/molK. These values are in good agreement with similar complexes of this type [8]. The temperatures of the peaks are almost identical to the values of the ST recorded with the magnetic measurements using a SQUID magnetometer.

## 3. Discussion

The general synthetic strategy of using rigid linkers to obtain cooperative spin transitions worked very nicely for the three different coordination polymers presented here. The synthesized complexes vary in the substituents of the equatorial ligand and in the amount of methanol molecules included in the crystal packing and therefore show a different SCO behavior. 

Complexes **1a** and **1b** only vary in the method of synthesis, but do not have the same magnetic properties and solvent content. As **1a** shows a half-complete ST with hysteresis and has no solvent included, whereas according to single-crystal XRD **1b** is already fully LS at 133 K and has two molecules of methanol included. Attempts to investigate the magnetic properties of **1b** were not successful as the methanol molecules were already lost during sample preparation and then exhibited the same properties as **1a**. Powder X-ray diffraction reveals different patterns for **1a** and **1b**, in line with the observed loss of the solvent. As the SCO properties of **1b** are unknown, the impact of those structural changes remains unclear. A change in the diffraction pattern upon solvent loss is also observed in the powder X-ray diffraction patterns of **3a** and **3b**. In the case of **3b** the polymer chains are separated by the methanol molecules; however, they are involved in several intermolecular hydrogen bonds and short contacts that give rise to the observed 73 K wide thermal hysteresis loop. The solvent-free complex **3a** has a 30 K wide hysteresis below room temperature. Powder X-ray diffraction of the compound in the HS and LS state indicates a phase transition during the ST. The hydrogen bond observed for the single crystals of **3b** between the ligand bpey and a carbonyl oxygen of the neighboring chain and the resulting pairing of two chains is solvent-independent. Therefore, this hydrogen bond is probably still present when the compound is dried. This would explain the magnetic behavior with hysteresis of **3a**, as the pairing of the chains leads to a higher cooperativity during spin crossover [66]. 

## 4. Materials and Methods

### 4.1. Experimental

All syntheses involving iron(II) were carried out under argon using Schlenk tube techniques. The used solvents were of analytical grade and degassed with argon for 30 min. The syntheses of the precursor complexes [FeL1/2/3(MeOH)_2_] were prepared as described in the literature [57,61,62,63]. The axial ligand 4,4′-dipyridylethylene was prepared according to the literature [64,65]. 

**[FeL1(bpey)]_n_** (**1a**). A dark red/brown solution of [FeL1(MeOH)_2_] (0.2 g, 0.448 mmol) and bpey (0.16 g, 0.888 mmol) in methanol (15 mL) was heated to reflux for one hour. After cooling and being left to stand at room temperature for 1 d, the purple powder was filtered off, washed twice with methanol (4 mL), and dried in vacuo to obtain **1a**. Yield: 0.17 g (67%). **MS** (EI (+)) *m*/*z* (%): 382 (C_18_H_18_FeN_2_O_4_, 46), 180 (C_12_H_8_N_2_, 100). Elemental analysis calculated for C_30_H_26_FeN_4_O_4_ (562.41 gmol^−1^_,_%): C 64.07, H 4.66, N 9.96; found C 63.82, H 4.76, N 9.95. 

**{[FeL1(bpey)]·2MeOH}_n_** (**1b**). Black crystals of **1b** were obtained by slow diffusion at room temperature of [FeL1(MeOH)_2_] (0.1 g, 0.224 mmol) and bpey (0.081 g, 0.450 mmol) in methanol solution after 1 week in a homemade Schlenk. 

**[FeL2(bpey)]_n_·1 MeOH** (**2**). A dark red/brown solution of [FeL2(MeOH)_2_] (0.2 g, 0.351 mmol) and bpey (0.13 g, 0.699 mmol) in methanol (15 mL) was heated to reflux for one hour. After cooling and being left to stand at room temperature for 3 d, the black powder was filtered off, washed with methanol (4 mL), and dried in vacuo to obtain **3**. Yield: 0.19 g (75 %). **MS** (EI (+)) *m*/*z* (%): 506 (C_28_H_22_FeN_2_O_4_, 100), 180 (C_12_H_8_N_2_). Elemental analysis calculated for C_40_H_30_FeN_4_O_4_·1 MeOH (718.59 gmol^−1^, %): C 68.53, H 4.77, N 7.80; found C 68.33, H 4.55, N 7.93. 

**[FeL3(bpey)]_n_·0.25 MeOH** (**3a**). A dark red/brown solution of [FeL3(MeOH)_2_] (0.2 g, 0.418 mmol) and bpey (0.151 g, 0.838 mmol) in methanol (15 mL) was heated to reflux for one hour. After cooling and being left to stand at room temperature for 2 d, the dark purple precipitate was filtered off, washed with methanol (5 mL) and dried in vacuo to obtain **3a**. Yield: 0.2 g (79%). **MS** (EI (+)) *m*/*z* (%): 414 (C_18_H_18_FeN_2_O_6_, 100), 180 (C_12_H_8_N_2_, 81). Elemental analysis calculated for C_30_H_26_FeN_4_O_6_·0.25 MeOH (602.42 g mol^−1^, %): C 60.31, H 4.52, N 9.30; found C 60.05, H 4.46, N 9.40.

**{[FeL3(bpey)]·2MeOH}_n_** (**3b**). Black crystals of **3b** were obtained by slow diffusion at room temperature of [FeL3(MeOH)_2_] (0.1 g, 0.210 mmol) and bpey (0.075 g, 0.416 mmol) in methanol solution after 2 weeks using a homemade Schlenk. 

### 4.2. X-ray Diffraction on Single Crystals

The X-ray analysis of **3b** was performed with a Stoe StadiVari diffractometer using graphite-monochromated MoKα radiation. The X-ray analysis of **1b** was performed with a Stoe IPDS II diffractometer using graphite-monochromated Mo-Kα radiation. The data were corrected for Lorentz and polarization effects. The structures were solved by direct methods (SIR-97) [75] and refined by fullmatrix least-square techniques against Fo2–Fc2 (SHELXL-97) [76,77]. All hydrogen atoms were calculated in idealized positions with fixed displacement parameters. ORTEP-III [78,79] was used for the structure representation, SCHAKAL-99 [80] was used to illustrate the molecule packing.

### 4.3. X-ray Powder Diffraction

Powder diffractograms were measured with a STOE StadiP Powder Diffractometer (STOE, Darmstadt) using Cu[Kα1] radiation with a Ge Monochromator and a Mythen 1K Stripdetector in transmission geometry.

### 4.4. Magnetic Measurements

Magnetic measurements on the compounds were carried out using a SQUID MPMS-XL5 from Quantum Design with an applied field of 5000 G, and in the temperature range from 400 to 50 K in settle mode. The sample was prepared in a gelatine capsule held in a plastic straw. The raw data were corrected for the diamagnetic part of the sample holder and the diamagnetism of the organic ligand using tabulated Pascal’s constants.

### 4.5. Mössbauer Spectrometry

^57^Fe Mössbauer spectra were recorded in transmission geometry at a constant acceleration using a conventional Mössbauer spectrometer with a 50 mCi ^57^Co(Rh) source. The spectra were fitted using the Recoil 1.05 Mössbauer Analysis Software [81]. The isomer shift values are given with respect to a α-Fe reference at room temperature.

## Figures and Tables

**Table 1:** Overview of all synthesized iron(II) complexes discussed in this work.

Compound	Complex	Method
**1a**	[FeL1(bpey)]_n_	reflux
**1b**	{[FeL1(bpey)]·2 MeOH}_n_	slow diffusion
**2**	{[FeL2(bpey)]·1 MeOH}_n_	reflux
**3a**	{[FeL3(bpey)]·0.25 MeOH}_n_	reflux
**3b**	{[FeL3(bpey)]·2 MeOH}_n_	slow diffusion

**Table 2:** Spin state, selected bond lengths/Å, and angles/° within the inner coordination sphere of **3b**, and angles and torsion angles of bpey.

Compound	*S*	Fe–N_eq_	Fe–O_eq_	Fe–N_ax_	O_eq_–Fe–O_eq_	N_ax_–Fe–N_ax_	bpey >C–C≡C	Torsion bpey
**3b**	0	1.898(4) 1.8921(4)	1.931(3) 1.931(3)	1.985(3) 1.997(3)	87.08(11)	175.18(14)	174.2(4) 176.9(4)	17.81 18.44

**Table 3:** Overview of the intermolecular interactions of **3b**.

Compound	D–H⋯A	D–H/Å	H⋯A/Å	D⋯A/Å	D–H⋯A/°
**3b**	O52–H52⋯O3	0.84	2.33	2.84(2)	119
	C26–H26⋯O3 ^a^	0.95	2.50	3.150(5)	126

a: 1−x, 2−y, 1−z.

**Table 4:** Overview of magnetic properties, *T*_1/2_ values, values of the *χ*_M_*T* product and the corresponding temperatures, and HS residues of the synthesized compounds. SCO: spin crossover.

	SCO	*T*_1/2_ [K]	*χ*_M_*T* (HS) [cm^3^Kmol^−1^]	*χ*_M_*T* (LS) [cm^3^Kmol^−1^]	γ_HS_
**1a**	Incomplete with hysteresis	↓ 175 ↑ 185	3.29 (250 K)	1.69 (50 K)	1 (250 K) 0.51 (50 K)
**2**	Stepwise, gradual with hysteresis	↓ 245 ↑ 260 ↓ 230 ↑ 240	3.17 (300 K)	0.09 (150 K)	1 (300 K) 0.03 (150 K)
	Stepwise, gradual with hysteresis ^a^	↓ 245 ↑ 260 ↓ 230 ↑ 240	3.23 (300 K)	0.20 (150 K)	1 (300 K) 0.06 (150 K)
**3a**	Abrupt with hysteresis	↓ 195 ↑ 225	3.31 (300 K)	0.19 (100 K)	1 (300 K) 0.06 (100 K)
**3b**	Abrupt with hysteresis	↓ 204 ↑ 277	3.79 (400 K)	0.08 (100 K)	1 (400 K) 0.02 (100 K)
	Abrupt with hysteresis ^a^	↓ 195 ↑ 225	3.79 (400 K)	0.12 (150 K)	1 (400 K) 0.03 (150 K)

a: after annealing.

## Supplementary Materials

The following are available online. Figure S1: ORTEP drawing of **3b** displaying the disorder of one substituent. Ellipsoids are drawn at a 50% probability level. Hydrogen atoms were omitted for clarity; Figure S2: Plots of the *χ*_M_*T* product versus *T* (sweep measurements) for compounds **1a**, **1b**, **2**, and **3a**; Figure S3: Mössbauer spectra of **1a**, **1b**, **2**, and **3a**; Table S1: Crystallographic data of **1b** and **3b**; Table S2: Mössbauer parameters of **1a**, **1b**, **2**, and **3a** at room temperature.
