# Supplementary material for: Iron(II) Spin Crossover Complexes with 4,4′-Dipyridylethyne—Crystal Structures and Spin Crossover with Hysteresis
Source: Molecules. 2020 Jan 29;25(3):581. doi: 10.3390/molecules25030581 (PMC7037042; doi:10.3390/molecules25030581)
Supplement: Supplementary file 1 [file molecules-25-00581-s001.pdf]

Supporting Information for

Iron(II) spin crossover complexes with 4,4'-dipyridylethyne – crystal structures and spin crossover with hysteresis

Katja Dankhoff[a], Charles Lochenie[a], and Birgit Weber[a]*

[a]: Lehrstuhl für Anorganische Chemie II, Universität Bayreuth, Universitätsstraße 30, NW I, 95440 Bayreuth, Germany. weber@uni-bayreuth.de

Table S1. Crystallographic data of **1b** and **3b**.

	**1b**	**3b**
formula	$C_{30}H_{26}FeN_4O_4 \cdot 2CH_3OH$	$C_{30}H_{26}FeN_4O_6 \cdot 2CH_3OH$
CCDC	1972794	1972795
M/ g mol^{-1}	626.48	658.49
crystal system	triclinic	triclinic
space group	*P1;*¯	*P1;*¯
a/ Å	11.2190(13)	11.8971(7)
b/ Å	11.1812(14)	12.3862(7)
c/ Å	11.8578(14)	12.5789(7)
α/ °	80.792(10)	66.929(4)
β/ °	79.902(9)	71.720(4)
γ/ °	75.533(9)	68.108(4)
V/ Å^3	1407.4(3)	1551.61(14)
Z	2	2
ρ_{calcd}/ g cm^{-3}	1.478	1.409
μ/ mm^{-1}	0.590	0.539
crystal size	0.052×0.313×0.064	0.098×0.381×0.094
F(000)	656	657
T/ K	140	133
λ/ Å	Mo-K$_\alpha$ 0.71073	Mo-K$_\alpha$ 0.71073
Θ range/ °	1.8–24.7	1.9–29.3
Reflections collected	16804	18736
independent reflections	4720	7756
reflections with $I \geq 2\sigma(I)$	1893	4510
R_{int}	0.294	0.064
reflections	4720	7756
parameters	391	396
R	0.0762	0.0716
$wR2$	0.1945	0.2053
S	0.79	0.93

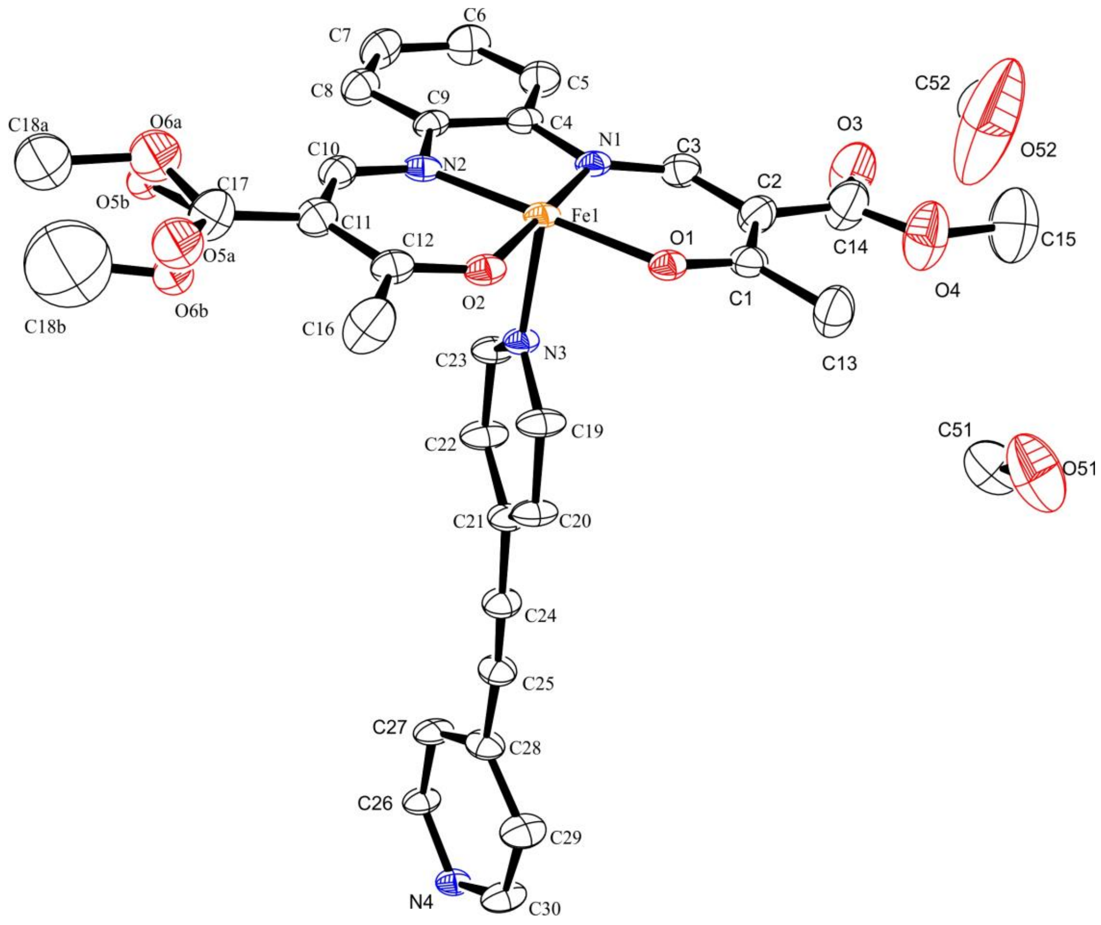

Figure S1. ORTEP drawing of **3b** displaying the disorder of one substituent. Ellipsoids are drawn at 50 % probability level. Hydrogen atoms were omitted for clarity.

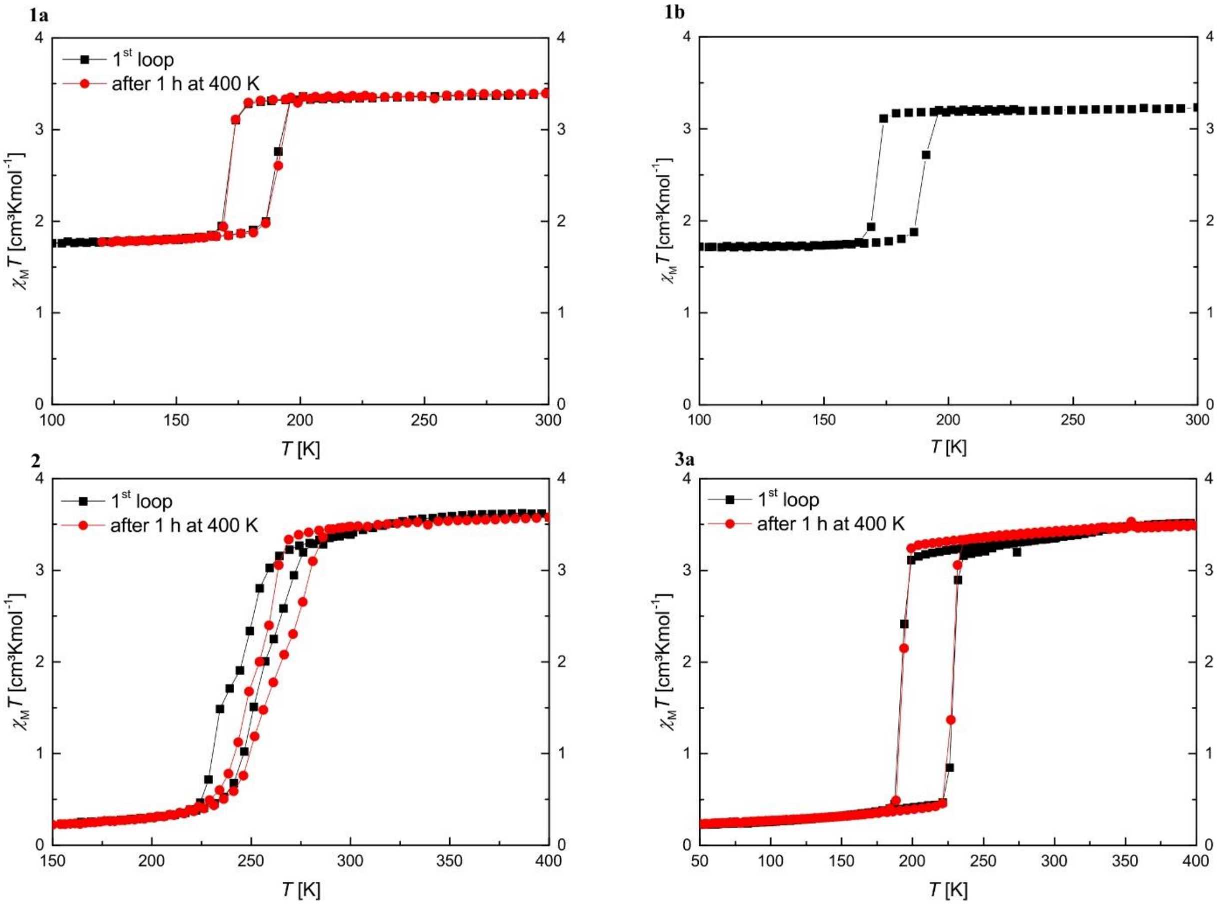

Figure S2. Plots of the $\chi_M T$ product versus T (sweep measurements) for compounds **1a**, **1b**, **2**, and **3a**.

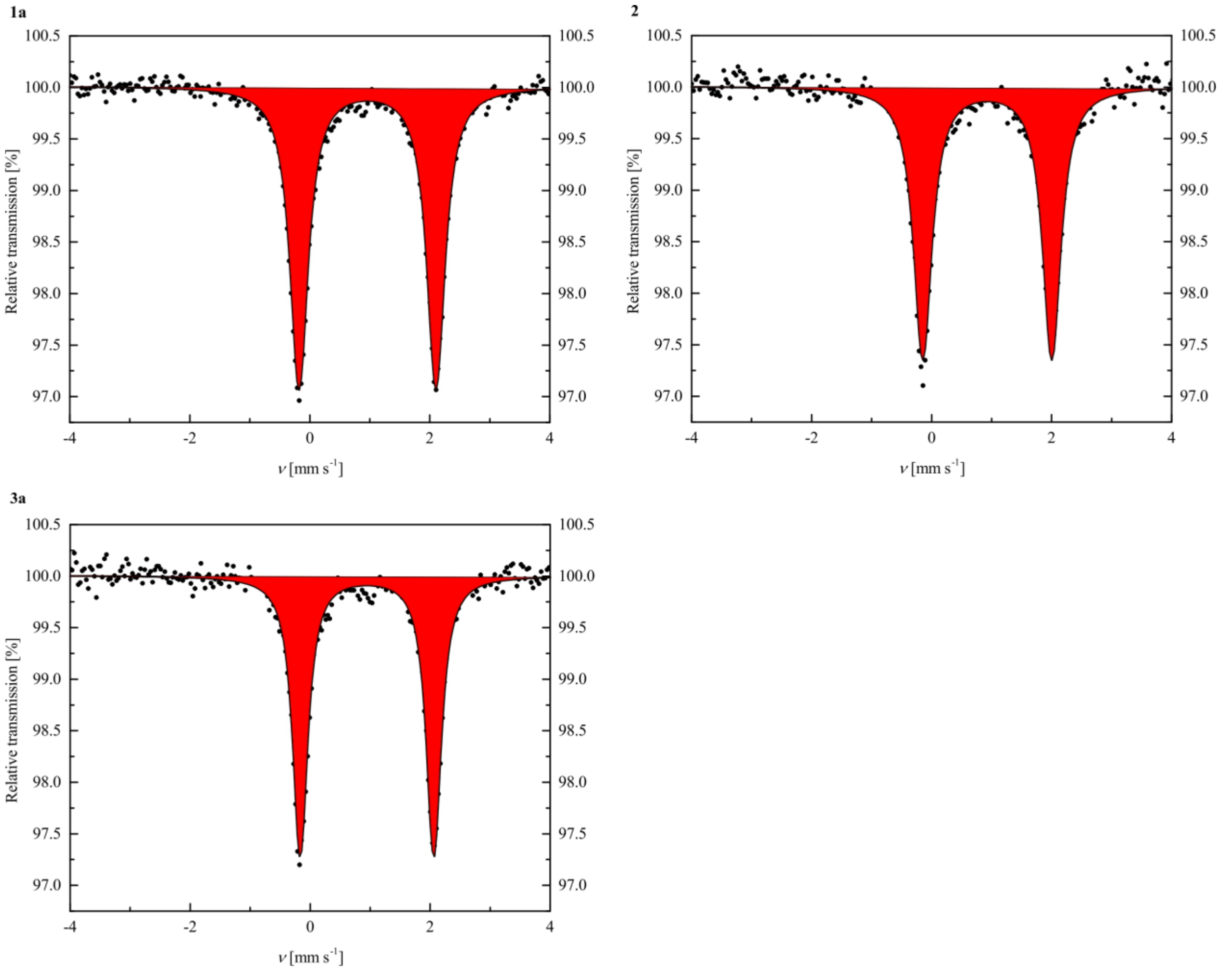

Figure S3. Mössbauer spectra of **1a**, **2**, and **3a**.

Table S2. Mössbauer parameters of **1a**, **2**, and **3a** at room temperature.

Sample	Site	Spin state	δ [mm s^{-1}]	ΔE_Q [mm s^{-1}]	$\Gamma/2$ [mm s^{-1}]
**1a**	D_1	HS	0.962(3)	2.281(7)	0.182(5)
**2**	D_1	HS	0.928(5)	2.151(10)	0.183(8)
**3a**	D_1	HS	0.948(4)	2.227(9)	0.150(7)
